# Supplementary figures and images for: The structural basis for deubiquitination by the fingerless USP-type effector TssM
Source: Life Sci Alliance. 2023 Dec 13;7(2):e202302422. doi: 10.26508/lsa.202302422 (PMC10719079; doi:10.26508/lsa.202302422)

B)

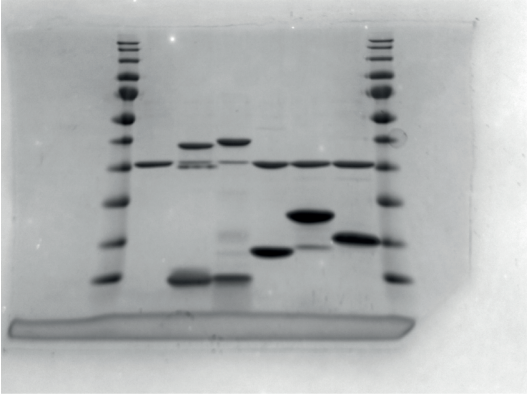

E)

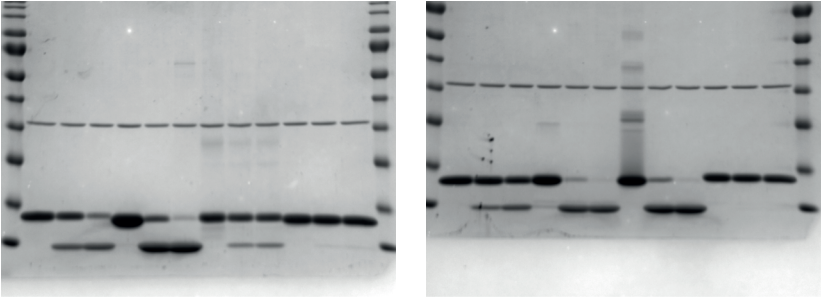

Supplement: Supplementary file 1 [file LSA-2023-02422_SdataF1.pdf]

B)

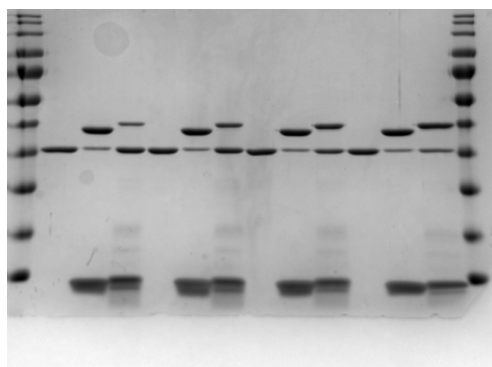

Supplement: Supplementary file 2 [file LSA-2023-02422_SdataFS1.pdf]

D)

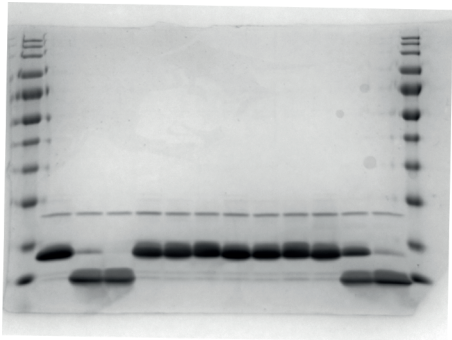

F)

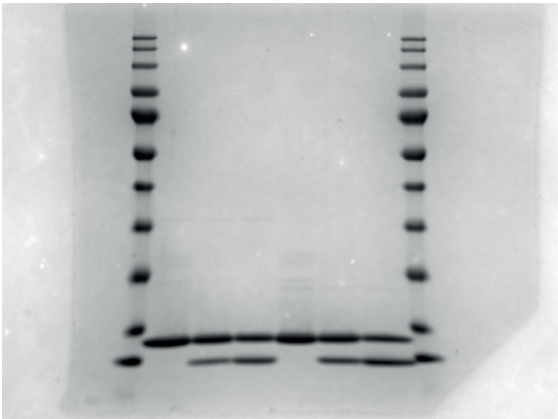

Supplement: Supplementary file 3 [file LSA-2023-02422_SdataF2.pdf]

G)

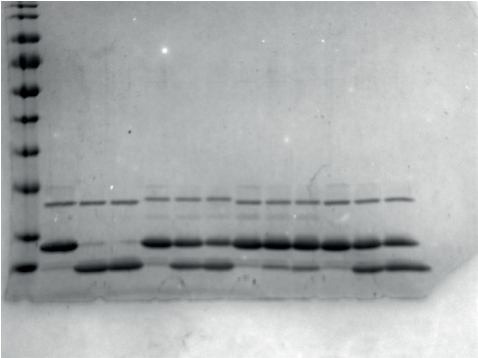

H)

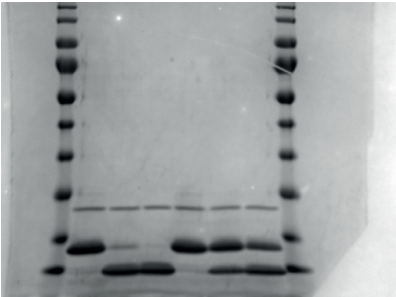

I)

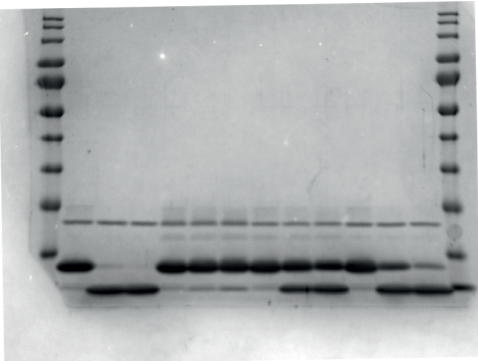

Supplement: Supplementary file 4 [file LSA-2023-02422_SdataF3.pdf]

1)

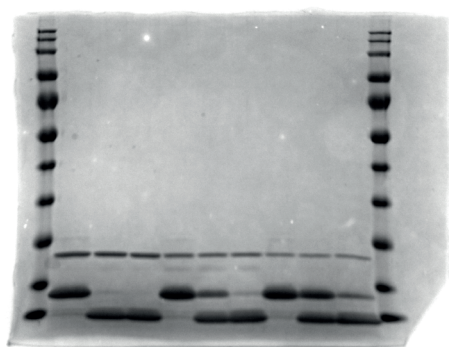

Supplement: Supplementary file 5 [file LSA-2023-02422_SdataFS3.pdf]
